# Large-Scale Expression Analysis Reveals Distinct MicroRNA Profiles at Different Stages of Human Neurodevelopment

**DOI:** 10.1371/journal.pone.0011109

**Published:** 2010-06-15

**Authors:** Brandon Smith, Julie Treadwell, Dongling Zhang, Dao Ly, Iain McKinnell, P. Roy Walker, Marianna Sikorska

**Affiliations:** Institute for Biological Sciences, National Research Council Canada, Ottawa, Canada; Deutsches Krebsforschungszentrum, Germany

## Abstract

**Background:**

MicroRNAs (miRNAs) are short non-coding RNAs predicted to regulate one third of protein coding genes via mRNA targeting. In conjunction with key transcription factors, such as the repressor *REST* (RE1 silencing transcription factor), miRNAs play crucial roles in neurogenesis, which requires a highly orchestrated program of gene expression to ensure the appropriate development and function of diverse neural cell types. Whilst previous studies have highlighted select groups of miRNAs during neural development, there remains a need for amenable models in which miRNA expression and function can be analyzed over the duration of neurogenesis.

**Principal Findings:**

We performed large-scale expression profiling of miRNAs in human NTera2/D1 (NT2) cells during retinoic acid (RA)-induced transition from progenitors to fully differentiated neural phenotypes. Our results revealed dynamic changes of miRNA patterns, resulting in distinct miRNA subsets that could be linked to specific neurodevelopmental stages. Moreover, the cell-type specific miRNA subsets were very similar in NT2-derived differentiated cells and human primary neurons and astrocytes. Further analysis identified miRNAs as putative regulators of *REST*, as well as candidate miRNAs targeted by *REST*. Finally, we confirmed the existence of two predicted miRNAs; pred-MIR191 and pred-MIR222 associated with *SLAIN1* and *FOXP2*, respectively, and provided some evidence of their potential co-regulation.

**Conclusions:**

In the present study, we demonstrate that regulation of miRNAs occurs in precise patterns indicative of their roles in cell fate commitment, progenitor expansion and differentiation into neurons and glia. Furthermore, the similarity between our NT2 system and primary human cells suggests their roles in molecular pathways critical for human *in vivo* neurogenesis.

## Introduction

miRNAs are small noncoding RNAs, identified through seminal work in *C. elegans* [1]–[3], *D. melanogaster* and humans [4] and are required for regulation of gene expression at the post-transcriptional level. To date, the miRBase release 14.0 miRNA registry (http://www.mirbase.org/) [5], [6] lists over 10,000 miRNAs, including 721 human miRNAs cloned and/or identified by homology to other organisms. It is further estimated that their total number in the human genome may exceed a thousand and they may target approximately one-third of all human messenger RNAs (mRNAs) [7].

Mature miRNAs of 18–22 nt in length are cleaved by the ribonuclease III family member Dicer from 60–90 nt hairpin-loop precursors (pre-miRNAs), and act to repress translation by binding with partial sequence complementarity to the 3′-UTRs of their target mRNAs. Depending in part on the characteristics of the mRNA promoter, miRNA∶mRNA binding results in inhibition of translation initiation or post-initiation translational block [8]. Each miRNA is predicted to recognize multiple target mRNAs [9], hence a single miRNA can have far-reaching effects on gene expression and may modulate the expression of gene sets that function together to control biological processes. This potential for coordinated control of gene expression, coupled with wide-ranging expression across multiple cell types and throughout development, implies that miRNAs regulate such cellular processes as stem cell self-renewal, fate determination and differentiation that are critical for normal development.

Several studies examine the expression of miRNAs during neurodevelopment as well as in the adult CNS and reveal that certain miRNAs are found preferentially expressed in neurons, e.g., miR-124 and miR-128 [10], whereas others, e.g., miR-23, miR-26 and miR-29, seem restricted to astrocytes [11]. Accordingly miRNAs have been implicated in many aspects of central nervous system development and function. For example, a recent report identifies miR-124 as a regulator of adult neurogenesis via the regulation of Sox9 in the subventricular zone stem cell niche [12]; whereas others highlight a role of miRNAs in brain morphogenesis [13], and neuronal cell specification [11], [14]–[16]. There are also reports linking non-coding RNAs with neurological disorders such as Parkinson's disease (miR-133b) [17], Huntington's disease (miR-132, miR-9) [18], [19], Alzheimer's disease (miR-29 and miR-107) [20], [21], and Tourette's syndrome (miR-189) [22].

The majority of existing studies examine a select number of miRNAs in the CNS and use PCR-based and/or Northern blotting techniques [10], [23]–[25]. A limited number of reports also present analysis of miRNA expression during neural differentiation of immortalized cell lines [26]–[28]. Sempere et al. [26] describe the expression of 66 miRNAs in human NTera2 and mouse P19 cells following retinoic acid (RA) treatment and establish that 19 of them are coordinately up-regulated in both cell lines indicating that their functions might be conserved between the species. In another study, Hohjoh and Fukushima [27] examine the profiles of 180 mouse and 127 human miRNAs in human (NTera2/D1) and rodent (P19D and Neuro2a) cell lines, and report that the ES cell specific miR-302 cluster [29], [30] and miR-124a [10] show the opposite patterns of expression in response to the RA treatment and may mark the onset of neural differentiation. In all tissues where it has been examined, it is apparent that miRNAs are subject to dynamic regulation during the process of development; however, to date, studies detailing miRNA expression in brain report small subsets of miRNAs and/or focus on specific tissues or sub-regions at restricted developmental time-points. As such these studies, although valuable, provide only a limited knowledge of the role of miRNAs in the process of neurogenesis as they are unable to capture the full dynamic profile of miRNA expression during neurogenesis and thus do not successfully recapitulate the developmental regulation of miRNAs in vivo. In particular, there are few studies that either extensively address miRNA expression throughout the complete duration of neurogenesis; or that define cell type-specific expression, such as the differences between fully differentiated neurons and glia.

To this end we undertook a large-scale microarray screening of miRNA expression during the process of neural differentiation in order to define expression patterns of miRNAs that can be linked to different stages of neurodevelopment and, subsequently, to specific pathways regulating neurogenesis. We used a human embryonal carcinoma (EC) cell line, NTera2/D1 (NT2), which is described as “a surrogate” of pluripotent embryonic stem cells that can undergo a multistep transition from the undifferentiated state to terminal neuronal differentiation. NT2, like pluripotent embryonic stem cells, have the capacity to differentiate into numerous cell types [31]. However when cultured at high-density under a 28 day treatment with RA they follow a highly reproducible neural differentiation program. RA-induced differentiation of NT2 cells proceeds through distinct phases of neural commitment, precursor expansion and terminal differentiation to neurons and astroglia [32] and analysis of neural gene markers shows a characteristic temporal and spatial pattern of gene expression similar to neuroepithelial precursors during *in vivo* neurogenesis [33], [34]. We performed extensive time-course analysis of 405 unique miRNAs during a four week RA-treatment of NT2 cells as well as in NT2-derived astrocytes (NT2-A) and NT2-derived post-mitotic neurons (NT2-N). Subsequently, we compared the data generated on NT2-derived cells with that of primary human neurons and astroglia. The large number of miRNAs analyzed, plus the duration and completeness of the time points examined significantly extend beyond those studies published previously. This is, to our knowledge, the first study of miRNA expression profiling extensively encompassing the duration of neurogenesis from surrogate human stem cells, through to neural precursors and in particular highlights the differences in miRNA expression of the terminally differentiated cell types (i.e., astroglia and post-mitotic neurons) originating from a common pool of progenitors. This is also the first comprehensive comparison of miRNA expression in NT2 neurons and astrocytes to their primary human counterparts. We report here the dynamic changes in miRNA expression during neural differentiation and the identification of distinct subsets of miRNAs in NT2 neurons and astrocytes, a pattern that is conserved in primary human cells.

## Results

### Microarray Platform and Data processing

We arrayed 405 miRNAs comprising 210 human and 49 mouse sequences and 146 computationally predicted miRNAs from Xie et al. [35], printed as trimeric concatemers. Previous use of multimeric concatemers in microarray probe design [36], [37] has revealed that dimer and trimer concatemers give stronger signals than monomers [37], and this approach has since found utility in commercial application (Invitrogen's nCode miRNA microarrays). Our preliminary studies confirmed the general increase in signal of multimeric concatemers over monomers, and also that trimers gave stronger signals than dimers without a significant loss of specificity (data not shown).

The microarray hybridizations were performed with Cy3-labeled, PEG enriched small RNAs collected during a time-course of RA- induced neural differentiation, i.e., from undifferentiated NT2/D1 ce1ls at day 0 (NT2-undiff), after 2, 4, 6, 8, 12, 14, 21 and 28 days of the RA treatment (NT2-2D…NT2-28D) and, subsequently from fully differentiated NT2-N neurons and NT2-A astrocytes. Our analysis also included a comparison between NT2-N neurons and NT2-A astrocytes and commercially available human primary fetal neurons (PHN), fetal astrocytes (PHAf) and embryonal astrocytes (PHAe). The complete set of expression data is presented in Fig. S1A and have been deposited in the Gene Expression Omnibus (GEO; http://www.ncbi.nlm.nih.gov/geo) under accession GSE15888.

The microarray data were normalized using a median centering procedure. This transformation resulted in a dataset that preserved both important characteristics in the frequency distributions of log2 intensity values (discussed below) and many large expression differences (both up- and down-regulation) of miRNAs in the RA time-course and in fully differentiated cells that were consistent with the literature.

**Figure 1:**
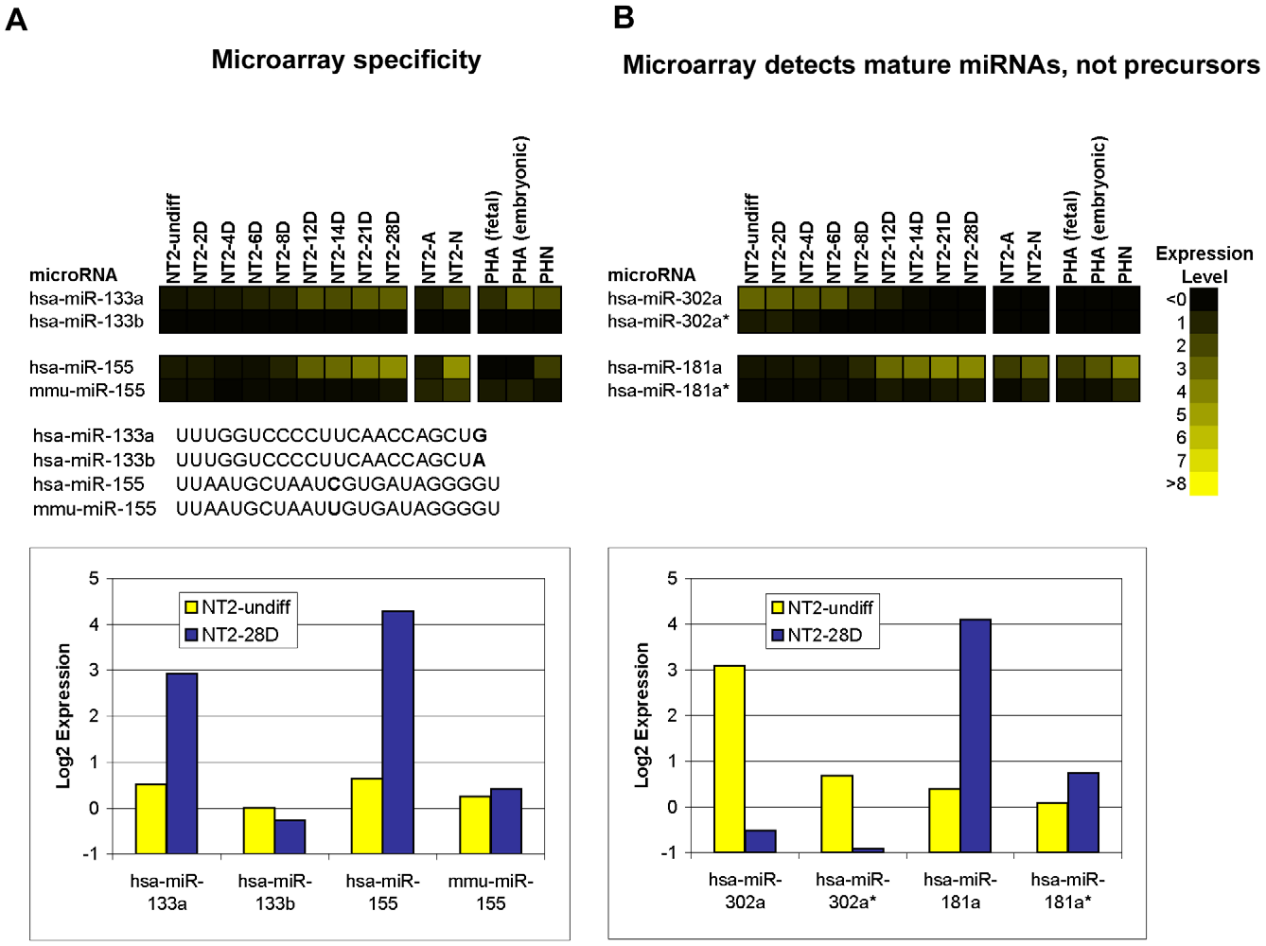
Specificity of microarray probes. (A) The expression patterns of miRNAs with single base differences during neural differentiation plotted as a heatmap (top). Sequence differences are shown in bold (middle). Comparison of their expression levels in NT2-undiff and NT2-28D plotted as yellow and blue bars, respectively (bottom). (B) The expression of major and minor mature miRNA pairs in the analyzed cells plotted as a heatmap (top). The expression levels of the miRNA pairs in NT2-undiff and NT2-28D plotted as yellow and blue bars, respectively (bottom). The intensity of the yellow scale in the heat map corresponds to the mean log2 expression of miRNAs on the microarrays, as shown in the key.

The specificity of the probes was demonstrated in hybridizations with RNA samples, in which we were able to unequivocally differentiate miRNAs with only single base differences, for example hsa-miR-133a and hsa-miR-133b, and the human and mouse forms of miR-155 (Fig. 1A). Furthermore, large differences in signal observed between major and minor products suggested that the microarray assay was detecting processed mature miRNAs rather than precursor miRNAs, which contain sequence for both major and minor products in a single RNA molecule (Fig. 1B). Consistently, the major mature miRNA product, miR-302a, was expressed strongly in undifferentiated NT2 cells, and down-regulated during the RA time-course while its minor counterpart, miR-302a*, was not expressed in any cell type. Similarly, major product, miR-181 was strongly up-regulated during RA treatment while the minor product, miR-181* was not detected in any cell type.

Microarray expression data was subjected to the following three thresholding criteria: (1) Minimum Intensity - miRNAs were considered expressed if their normalized log2 intensity was ≥2 in at least one time point; (2) Statistical Significance – miRNA expression changes were identified using a SAM-FDR threshold of 5%; and (3) Differential Expression - a minimum 2-fold difference in either direction was required.

**Figure 2:**
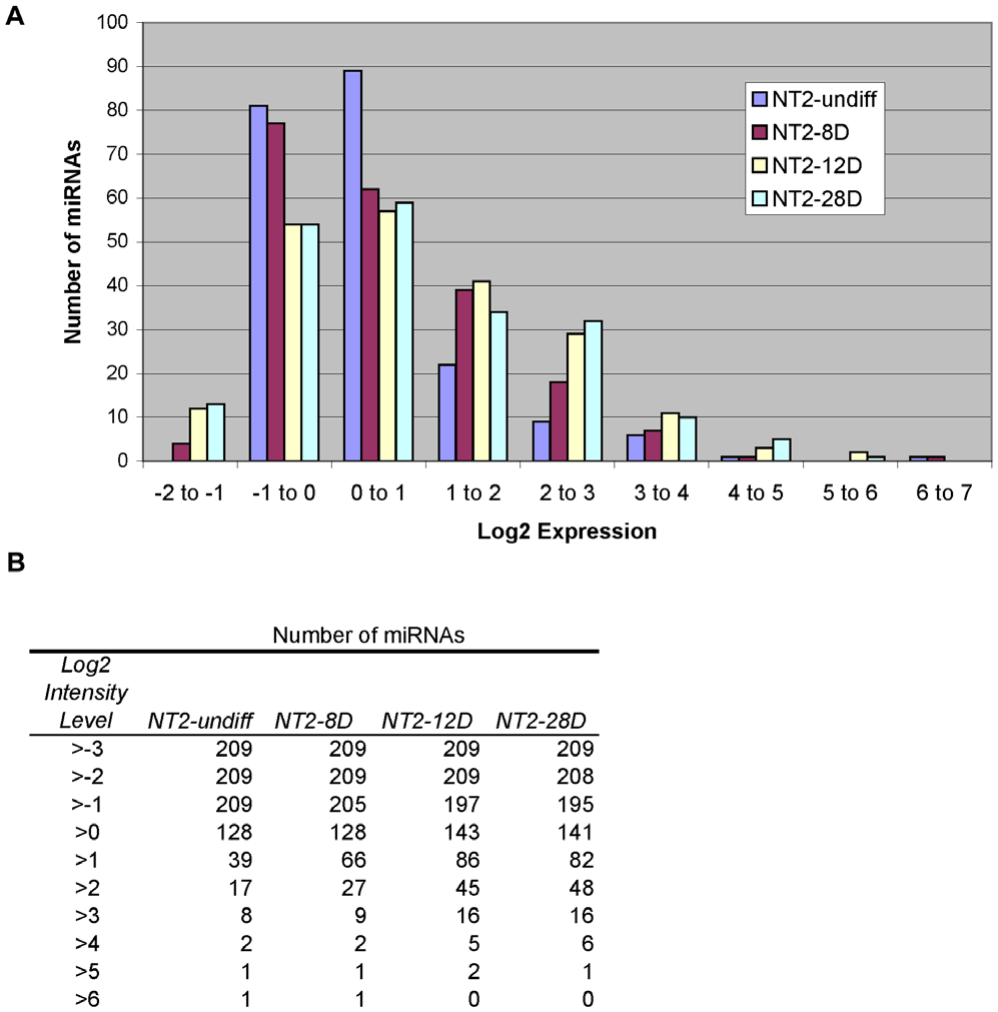
Distribution of human miRNAs according to microarray intensity level. (A) A histogram showing the distribution of the log2 intensity values of human miRNAs in NT2-undiff, NT2-8D and NT2-28D. (B) The data from A is tabulated to show the number of miRNAs with log2 intensity equal to or greater than specific values.

We observed that, as the RA time-course progressed, the number of miRNAs with higher intensity increased. Fig. 2A shows the frequency distribution of the log2 intensity of human miRNAs at various time points in the NT2-RA time-course. For all time points the log2 intensity of the majority of miRNAs was in the −1 to 0 and 0 to 1 ranges (Fig. 2A), however, as the time-course progressed there was an increasing number of miRNAs with log2 intensity levels >1 (39, 66, and 82 miRNAs in NT2-undiff, NT2-8D and NT2-28D, respectively; Fig. 2B). The shift in the distribution was more striking at higher intensity: almost three times as many miRNAs had log2 intensity levels >2 in NT2-28D than NT2-undiff (48 and 17, respectively; Fig. 2B).

**Figure 3:**
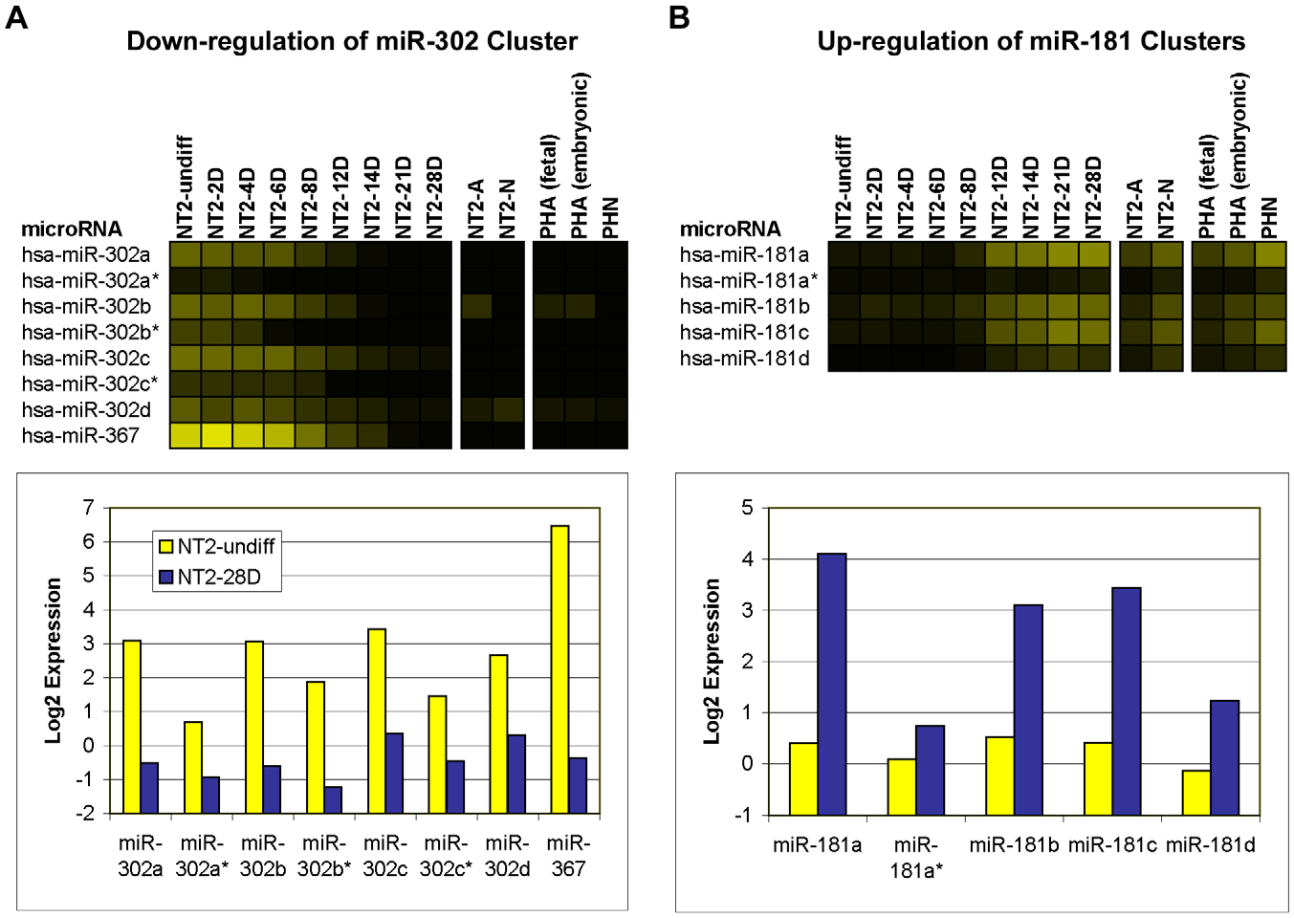
Microarray sensitivity. The expression levels of the members of the miR-302 (A) and the miR-181 (B) genomic clusters during neural differentiation plotted as heatmaps (top panels). The expression levels of the same miR-302 (A; bottom left panel) and miR-181 (B; bottom right panel) genomic clusters in NT2-undiff and NT2-28D plotted as yellow and blue bars, respectively. The intensity of the yellow scale in the heat map corresponds to the mean log2 expression of miRNAs on the microarrays, as shown in the key on Figure 1.

In many cases we observed that related miRNAs, for example major and minor products of the same precursor, and miRNAs expressed in a polycistronic cluster, showed similar expression profiles even though some members had log2 intensity below threshold. For example, the polycistronic miR-302 cluster was found to be strongly down-regulated during the RA time-course (Fig. 3A). All of the miRNAs in this cluster passed the significance and fold-change thresholds for down-regulation at 28 days RA, however all of the minor products (indicated in mirBase with an asterisk after the name) had log2 intensity below threshold (Fig. 3A). Similarly, miR-181 family members, which are located in three genomic clusters: mir-181a-1 and mir-181b-1 on chromosome 1; mir-181a-2 and mir-181b-2 on chromosome 9; and mir-181c and mir-181d on chromosome 19, were all up-regulated during the RA time-course, but miR-181a* and miR-181d had log2 intensity below threshold at 28 days RA (Fig. 3B). The observed increasing trend in the number of miRNAs with log2 intensity >1 through the RA time-course and the numerous examples of miRNAs with low intensity values that show agreement in expression profiles with other cluster members, demonstrated the ability of the microarray to detect differences at low intensity. However, in our analysis we applied a more conservative minimum intensity threshold (i.e. log2 intensity ≥2) in order to reduce the potential for false positives and focus on miRNAs that could be said to be expressed with a high degree of confidence. Applying this threshold in our study, 47 percent of the human miRNAs on the microarray (99 of 210) were considered expressed in at least one cell type.

### miRNA expression profiles during the time-course of RA treatment of NT2 cells (neurogenesis)

During RA-induced neurogenesis NT2 cells transition through distinct temporal phases, beginning with stem cell to precursor expansion, neural commitment and onset of cell-cycle exit (NT2-undiff - NT2-8D), cell cycle exit and onset of neural differentiation (NT2-8D–NT2-12D) and terminal differentiation (NT2-12D–NT2-28D) to neurons and astroglia (NT2-N, NT2-A); hence our data analyses focused on these time points.

**Figure 4:**
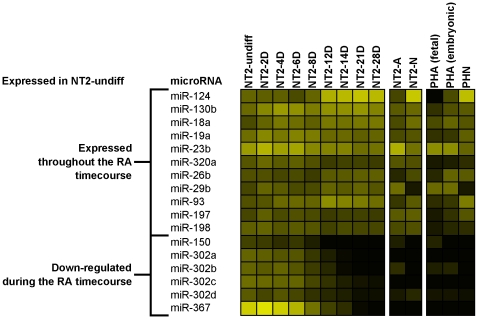
Profiles of human miRNAs expressed in the stem cell state of NT2 cells (NT2-undiff). The intensity of the yellow scale in the heat map corresponds to the mean log2 expression of miRNAs on the microarrays, as shown in the key on Figure 1.

Initially, only 17 human miRNAs were significantly expressed in the stem cell state (NT2-undiff; Fig. 4), of which 11 remained expressed throughout the RA treatment and subsequently in one or both neural cell types. Five of the other 6 miRNAs were members of a known ES cell specific polycistronic cluster (miR-367, miR-302a, b, c and d) and one, miR-150, which was only weakly expressed in NT2-undiff, were down-regulated during the RA time-course (Fig. 4). Additionally, three minor products of the polycistronic cluster (miR-302a*, miR-302b* and miR-302c*) were also down-regulated, but were expressed below threshold in NT2-undiff (Fig. 3B). Given the phasic nature of NT2 neurogenesis, we performed three differential expression analyses at specific time-points of the NT2 RA treatment in order to better characterize the miRNA expression profiles at these phases. Thus, the comparison was made between undifferentiated state and day 8 of RA (NT2-undiff vs. NT2-8D), day 8 and 12 of RA (NT2-8D vs. NT2-12D), and undifferentiated state and day 28 of RA (NT2-undiff vs. NT2-28D). miRNAs meeting 3 criteria for differential expression (expression ≥2, SAM-FDR≤5% and fold-change ≥2) were classified as up- or down-regulated with respect to the first condition and are listed in Fig. S1B.

### Precursor expansion, neural commitment and onset of cell cycle exit

**Figure 5:**
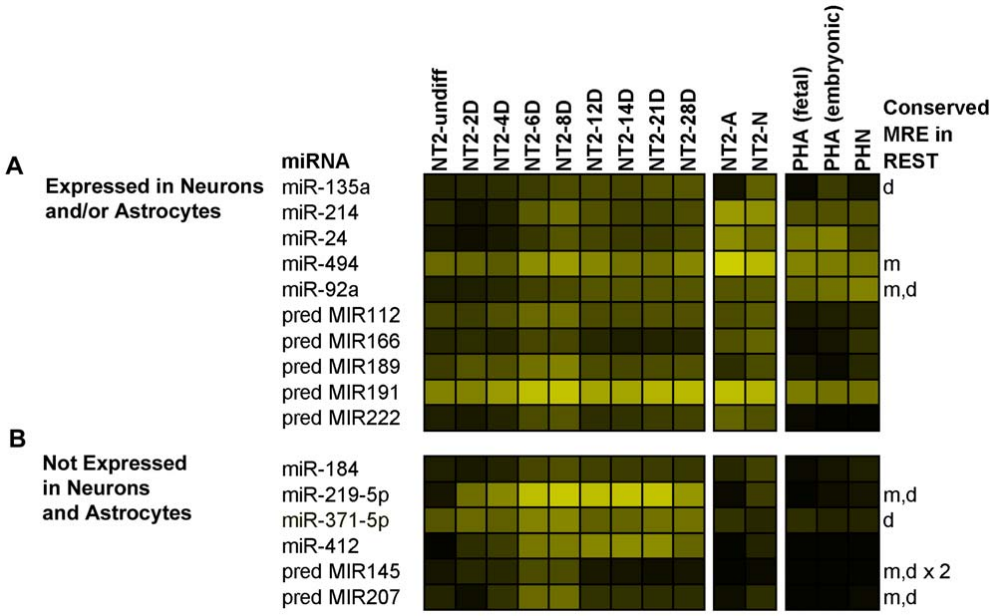
Expression profiles of miRNAs induced within the first 8 days of the RA treatment. (A) Subset of miRNAs induced during 8 days of RA treatment and subsequently expressed in NT2-derived differentiated cells. (B) Subset of miRNAs expressed transiently during the RA time-course, but not detected in either NT2-derived or human primary differentiated cells. Indicated are miRNAs for which MREs were found in the 3′UTR of human *REST* and conserved in mouse (m), and/or dog (d), ×2 indicates that 2 conserved MREs were found. The intensity of the yellow scale in the heat map corresponds to the mean log2 expression of miRNAs on the microarrays, as shown in the key on Figure 1.

**Figure 6:**
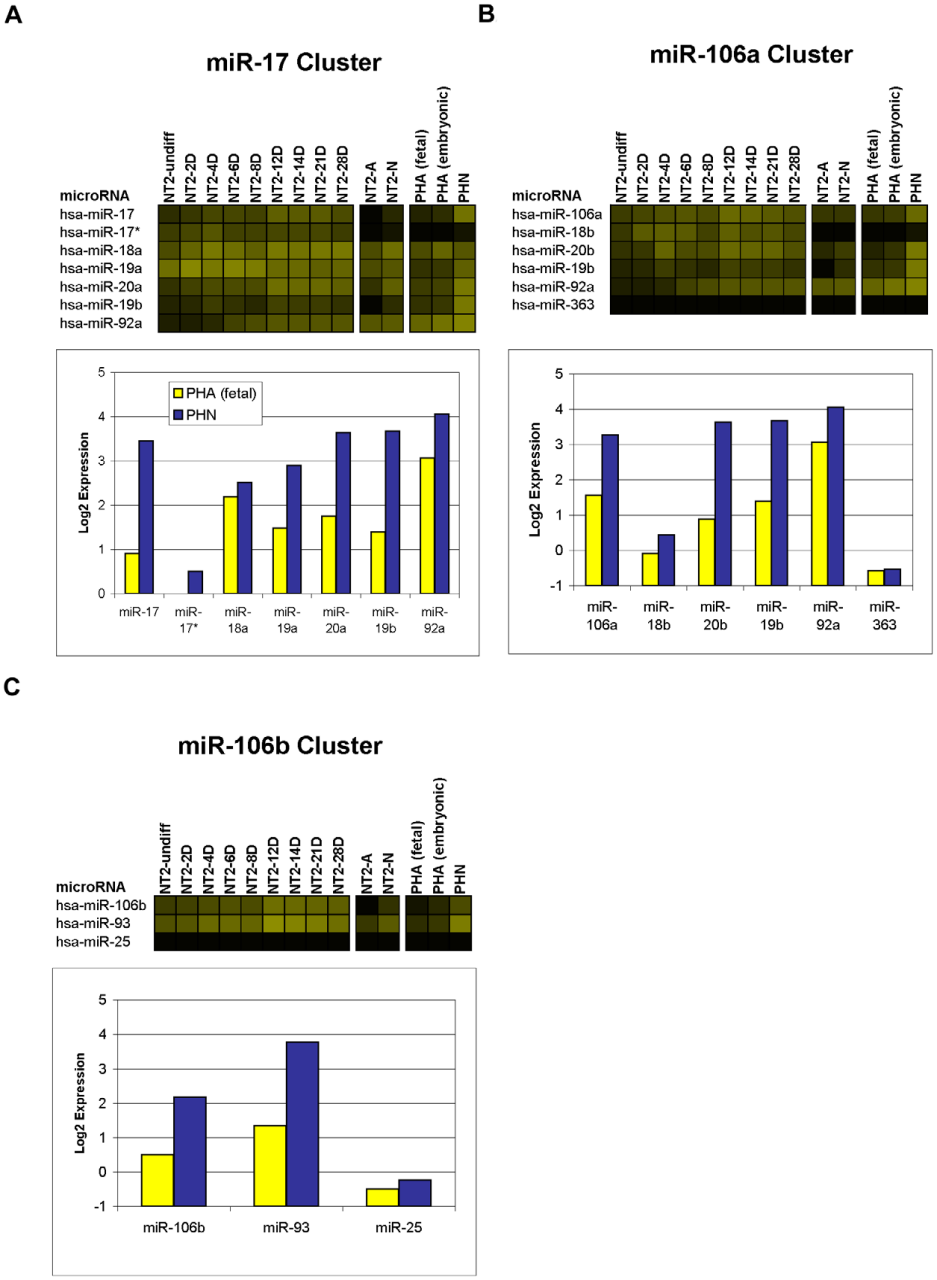
Expression of neuronal miRNAs in genomic clusters. Expression of 3 related genomic clusters containing predominantly neuronal miRNAs; the miR-17 cluster (A), the miR-106a cluster (B) and the miR-106b cluster (C). Each cluster is plotted as a heatmap; and as a bar graph which shows the expression level of the miRNAs in PHAf and PHN plotted in yellow and blue, respectively. The intensity of the yellow scale in the heat map corresponds to the mean log2 expression of miRNAs on the microarrays, as shown in the key on Figure 1.

**Figure 7:**
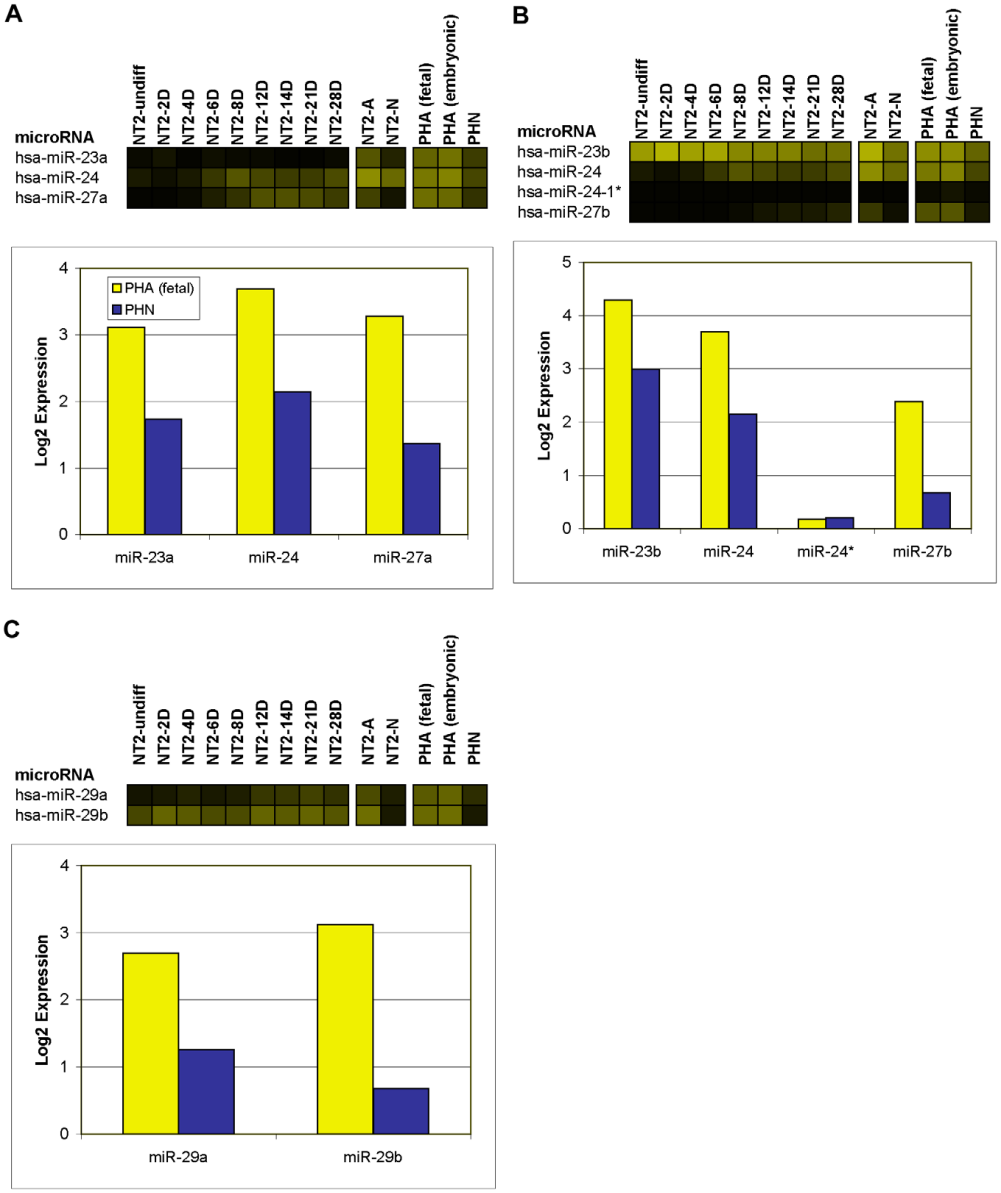
Expression of astrocytic miRNAs in genomic clusters. Expression of 3 genomic clusters containing predominantly astrocytic miRNAs; the miR-23a cluster (A), the miR-23b cluster (B) and the miR-29a cluster (C). Each cluster is plotted as a heatmap; and as a bar graph which shows the expression level of the miRNAs in PHAf and PHN plotted in yellow and blue, respectively. The intensity of the yellow scale in the heat map corresponds to the mean log2 expression of miRNAs on the microarrays, as shown in the key on Figure 1.

Of particular interest were 16 miRNAs that were induced within the first 8 days of RA (Fig. 5). During this phase there is an initial increase in expression of the intermediate filament protein nestin (*NES*), peaking at 3 days of RA, indicative of a temporary pool of proliferating neuroprogenitor cells [32]. Nestin is observed to drop off around 6–7 days of RA treatment, with a concurrent increase in the expression of the transcription factor neurogenic differentiation 1 (*NEUROD1*) characterizing the onset of exit from the cell-cycle [32]. With the exception of miR-135a, which increased throughout the RA time-course, these miRNAs showed a similar pattern of expression, i.e., in addition to being clearly up-regulated during early stages of neural differentiation, their expression peaked between day 6 and day 14 of RA (Fig. 5 and Fig. S1B). Six of these miRNAs, i.e., miR-184, miR-371-5p, miR-412, miR-219-5p, pred-MIR145 and pred-MIR207 were expressed only during the RA time-course (Fig. 5B). Interestingly, the 2 predicted miRNAs (pred-MIR145 and pred-MIR207) were expressed only transiently during the RA time-course, being detected only at days 6 and 8 of the treatment, potentially explaining why these miRNAs have not yet been cloned (Fig. 5B). The remaining 10 miRNAs, including 5 known (miRNAs 24, 92a, 135a, 214, and 494) and 5 predicted (pred-MIR112, pred-MIR166, pred-MIR189, pred-MIR191, and pred-MIR222) continued to be expressed in fully differentiated NT2-N neurons and/or NT2-A astrocytes (Fig. 5A). Notably, of the 5 known miRNAs, miR-92a and miR-24 are both members of described genomic miRNA clusters. Paralogs of miR-92a, are located in the miR-17 and miR-106a genomic clusters, which we found to be expressed predominantly in neurons, both in NT2-N and in primary human neurons (microarray data validated by qPCR; Fig. 6A, B & Table S1). Similarly, paralogs of miR-24 occur in the miR-23a and miR-23b genomic clusters that were detected in NT-A and primary human astrocytes (microarrays and/or qPCR; Fig. 7A, B & Table S1).

One of the key steps in neurogenesis is the down-regulation of the transcriptional repressor, RE1 silencing transcription factor (*REST*), which represses neuronal mRNAs in non-neuronal cells, including undifferentiated neural progenitors [38], [39]; subsequent loss of *REST* repression then drives cells towards commitment to a neural phenotype. As such, miRNAs up-regulated in the first 8 days of RA, just prior to the down-regulation of *REST* [34], are good candidates as *REST* targeting miRNAs. Using miRanda [40] we searched the 3′-UTR of human, mouse and dog *REST* for conserved putative miRNA recognition elements (MREs) of the subset of miRNAs up-regulated in the first 8 days of RA treatment. We identified MREs for two miRNAs conserved in human and dog (miR-135a and miR-371-5p), one conserved in human and mouse (miR-494) and four conserved in human, mouse and dog (miR-92a, miR-219-5p, pred-MIR145 and pred-MIR207; Fig. 5). The miR-219-5p MRE was of particular interest as this miRNA was immediately and robustly up-regulated on addition of RA. At 2, 4, 6 and 8 days RA versus NT2-undiff, miR-219-5p showed 7, 12, 40 and 54-fold up-regulation, respectively. Furthermore, from Day 6 through Day 14, this miRNA showed the highest intensity of all miRNAs on the array. The identification of these elements, together with their conservation across species and described expression dynamics, suggest these miRNAs may well be good candidates for the regulation of *REST* expression.

### Cell-cycle exit and onset of neural differentiation

**Figure 8:**
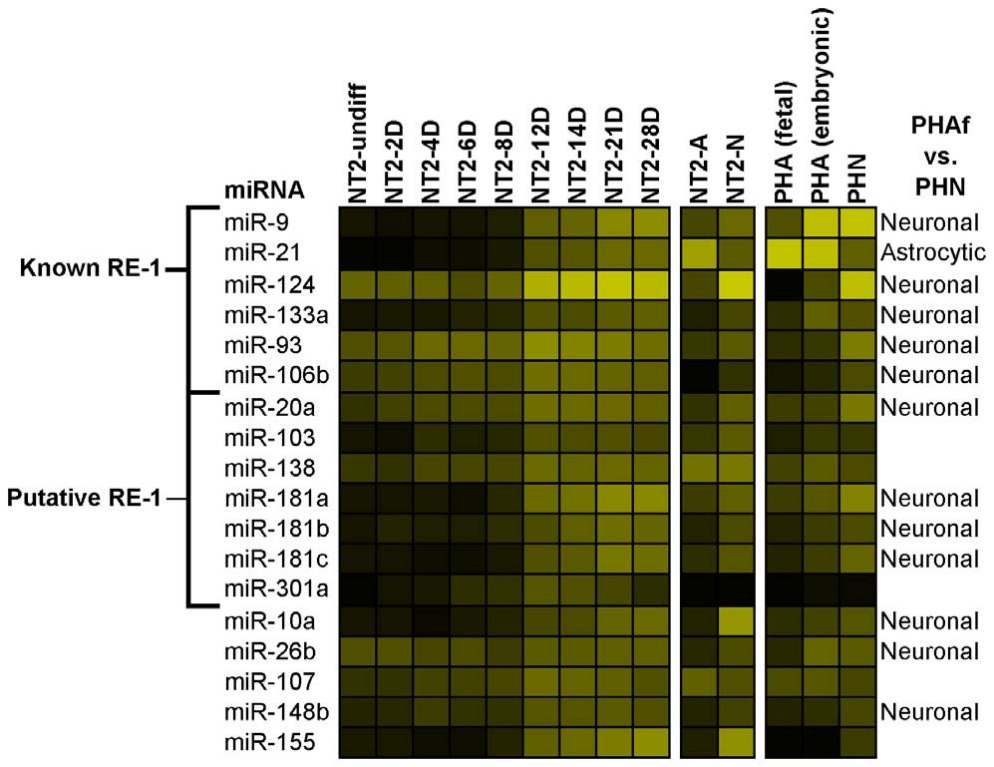
Expression profiles of miRNAs induced between 8 and 12 days of the RA treatment. Subset of miRNAs induced between 8 and 12 days of RA treatment. Indicated are miRNAs containing known or putative RE-1 sites and miRNAs labeled as Neuronal or Astrocytic based on the comparison between human primary neurons with primary astrocytes (PHN vs. PHAf). The intensity of the yellow scale in the heat map corresponds to the mean log2 expression of miRNAs on the microarrays, as shown in the key on Figure 1.

Between 8 and 12 days of the NT2 RA time-course nestin expression continues to decline whilst *NEUROD1* is robustly expressed [32]. By the end of this period most cells have exited the cell cycle and are committed to differentiate to a neural phenotype, as evidenced by a drop in the percentage of BrdU and Ki67 positive cells and the emergence of MAP2 positive cells [32], [33]. In these cells, this phase follows the down-regulation of *REST*, which has been shown to repress transcription of a number of neural miRNAs including miR-124, miR-9, miR-21, miR-106b and miR-93 [16], [41]. During this phase 18 miRNAs were up-regulated (Fig. 8), including the above mentioned *REST* target miRNAs and miR-133a, a likely *REST* target that is up-regulated during differentiation of human HSF6 ESCs to neurons [42], [43]. In addition we identified 7 putative *REST* repressed genes by their proximity to predicted and/or chromatin immunoprecipitated (ChIP) RE-1 elements (Table 1). Interestingly, most of the miRNAs differentially expressed between 8 and 12 days RA were also found to be differentially expressed in primary human neurons and astrocytes, including all 6 known, and 4 putative *REST* regulated miRNAs; miR-21 was astrocytic and the other 9 were neuronal (Fig. 8). Furthermore, while some of these miRNAs, including miR-124, were expressed above threshold earlier in the time-course, none of them were significantly induced within the first 8 days of RA suggesting that loss of *REST* may be required for induction of these miRNAs.

**Table 1:** RE-1 sites near miRNAs.

miRNA	RE-1distance (kbp)	HumanChIP-Seq	Score inRE-1db
miR-9-1	−238.0	Yes	0.962
	5.7	Yes	0.9628
mir-9-2	213.8	Yes	0.9523
	213.9	Yes	0.8925
miR-9-3	3.0	Yes	0.9426
	6.1	Yes	
miR-21	298.6	Yes	
miR-124-1	−46.6	Yes	
	−21.7	Yes	0.9237
miR-124-2	0.8	Yes	0.981
	−108.5	No	0.9357
	−61.9	No	0.9645
	−2.4	Yes	0.9108
mir-124-3	0.4	No	0.9583
	200.2	Yes	0.8986
	283.0	No	0.9177
	474.7	No	0.9223
	−333.1	Yes	0.9584
miR-1-1/miR-133a-2	12.0	Yes	
	22.9	No	0.9398
miR-93/106b		No	
miR-20a (miR-17 cluster)	292.3	Yes	0.9095
miR-103-1	32.0	Yes	0.9364
miR-103-2	−130.0	Yes	
miR-138-1	22.3	Yes	
	418.7	Yes	0.928
miR-181a-2/b-2	−276.8	Yes	
	77.5	Yes	
miR-181c/d	−433.0	Yes	0.9669
	−411.0	Yes	0.9645
miR-301a	239.4	No	0.9128
	395.0	Yes	

Known and putative RE-1 sites regulating miRNAs were identified by aligning the genomic locations of human miRNAs from miRBase, obtained from the UCSC human genome browser (hg17) sno/miRNA track, with putative REST sites from the human RE-1db [56] and data from a ChIP-Seq analysis for REST in human K562 cells [42]. RE-1 sites were associated with a miRNA if they were less than 500kbp away and found either in the ChiP-Seq data or in RE-1db mkII with a PSSM score greater than 0.91.

### Neuronal and astrocytic miRNA profiles (terminal differentiation)

At 28 days of RA treatment the NT2 cell cultures contained both neurons and astrocytes and the miRNA expression pattern at this time point represented this mixed population of cells. In order to establish the cell-type specific patterns we performed miRNA expression profiling on cultures of pure NT2-N neurons expressing neuron-specific enolase (*ENO2*) and NT2-A astrocytes expressing *GFAP* produced using a protocol established in our laboratory [32], [44]. Using the same criteria as for the RA treatment time-course (expression ≥2, SAM-FDR≤5% and fold-change ≥2), miRNA expression of the pure cell populations was compared to 28 Day RA treated NT2 cells (NT2-28D vs. NT2-N and NT2-28D vs. NT2-A) and miRNAs meeting these criteria were classified as higher or lower with respect to NT2-28D. We also compared the cells to each other (NT2-N vs. NT2-A) and classified the miRNAs as neuronal or astrocytic based on their relative expression. The data is summarized in Fig. S1C.

The comparisons revealed that the expression of 26 miRNAs was higher in NT2-N as compared to NT2-28D of RA, whereas 13 miRNAs showed a lower expression level. The opposite trend was observed in NT2-A in which the expression of 40 miRNAs was lower and only 13 was higher in comparison to NT2-28D. We found that the expression of miRNAs labeled as astrocytic (from the NT2-A vs. NT2-N comparison) was in all cases but one, higher in NT2-A than NT2-28D, whereas the expression of the majority of neuronal miRNAs (13 of 17) was lower in NT2-A compared to NT2-28D. In general, all miRNAs classified as neuronal or astrocytic in the NT2-N vs. NT2-A comparison were also differentially expressed in one, or both differentiated cells data sets in comparison with NT2-28D (Fig. S1C). Interestingly, 15 of 17 miRNAs labeled neuronal, but only 1 (miR-21) of 10 astrocytic were up-regulated during the 28 Day RA time-course (Fig. S1A).

### Comparison between primary and NT2-derived human neurons and astrocytes

To further characterize the miRNA profiles of human neurons and astroglia we performed 2 comparative analyses of differential expression in PHAf vs. PHN and PHAe vs. PHN using the same criteria as for the time-course of NT2 cells RA treatment. These data sets were subsequently compared with the NT2-A vs. NT2-N set. The miRNAs meeting all criteria (expression ≥2, SAM-FDR≤5% and fold-change ≥2) were labeled as astrocytic or neuronal based on their relative expression. The data is summarized in Fig. S1D.

We identified 49 neuronal and 14 astrocytic miRNAs from the comparison of their expression in PHAf vs. PHN. Of these, 21 neuronal and 10 astrocytic miRNAs met the screening criteria in PHAe vs. PHN, and 11 neuronal and 10 astrocytic in NT2-A vs. NT2-N. Four miRNAs (miR-27a, miR-27b, miR-24, and miR-29a), although classified astrocytic in primary cells, passed the ratio threshold, but failed either the expression level or SAM-FDR threshold criteria in NT2-A vs. NT2-N. The astrocytic expression of miR-24 and miR-27a, however, was confirmed by qPCR (Table S1). miR-24 and miR-27a are members of a genomic cluster on chromosome 19 with the astrocytic miRNA miR-23a (Fig. 7A). A paralogous miR-24 gene is found on chromosome 9 with miR-27b and the astrocytic miRNA miR-23b (Fig. 7B). miR-29a is clustered with the astrocytic miRNA miR-29b on chromosome 7 (Fig. 7C). This suggested that members of these clusters are astrocytic in both NT2-A and primary human cells.

Of the 49 neuronal miRNAs identified in primary human neurons, 27 passed the ratio threshold in NT2-N vs. NT2-A and similarly 12 of 17 NT2 neuronal miRNAs passed the ratio threshold in PHN vs. PHAf cells. Interestingly, all 49 neuronal miRNAs were more strongly expressed in PHAe than in PHAf. Among the 49 neuronal miRNAs in primary human neurons were members of 3 related genomic clusters; 4 members of the miR-17 cluster on chromosome 13 (miRNAs 17, 19a, 19b, and 20a; Fig. 6A), 3 members of the miR-106a cluster on chromosome X (miRNAs 19b, 20b, and 106a; Fig. 6B) and 2 members of the miR-106b cluster on chromosome 7 (miRNAs 106b and 93; Fig. 6C). All of the members of these clusters showed higher expression in PHN than PHAf (Fig. 6A,B,C). In general, not all astrocytic and neuronal miRNAs (classified based on their expression in primary cultures) were expressed at 28 days RA treatment of NT2 cells, but most of them were expressed in pure populations of NT2-A (93%) and NT2-N (63%) cells (Fig. S1A). Thus, for the most part, NT2-A astrocytic miRNAs were also astrocytic in primary human cells and, similarly, miRNAs labeled neuronal in NT2-N were also neuronal in their primary human counterparts.

### Validation of microarray data by qPCR

Validation of the microarray results for known miRNAs was carried out by real-time qPCR using a TaqMan miRNA assay. qPCR was performed on a selection of the miRNAs identified as differentially expressed by microarrays (from comparisons of undifferentiated NT2 cells at day 0 with those at day 8, 14, 21 or 28 of RA treatment; primary human neurons and fetal astrocytes; primary human neurons and embryonic astrocytes; and NT2 neurons and astrocytes). Additionally, a number of miRNAs that failed one or more threshold criteria on the microarrays were also examined by qPCR and were found to be differentially expressed. Overall over 90 percent of the miRNA microarray results tested were validated by qPCR, confirming the integrity of the data generated by the microarray platform. Extensive validation details are provided in Table S1.

### Identification of predicted miRNAs by qPCR and amplicon sequencing

**Figure 9:**
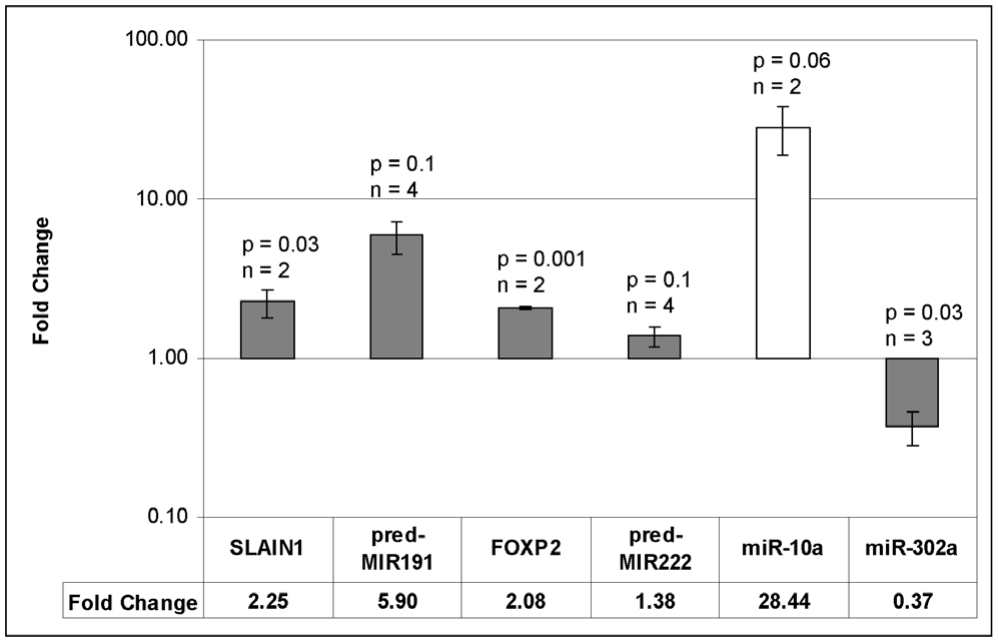
Real-time qPCR analysis of predicted miRNAs and associated host gene mRNAs. Fold change values are means of n replicates (n as indicated), and p-values are derived from 2-tailed student's t-tests. Error bars are derived from the S.E.M. of the ΔCT values (average fold change×(2^SEM^−1)). The grey bars are NT2-8D/NT2-undiff and the white bar is NT2-28D/NT2-undiff. As there was no amplification of miR-10a in NT2-undiff a value of 40 cycles was used to compute the ΔCT values.

Since this study was undertaken a number of the predicted miRNAs from Xie et al. [35] have subsequently been confirmed by others and already appear in miRBase (indicated in Table S1A), but many still remain unidentified. We selected 6 predicted miRNAs (pred-MIR112, 145, 189, 191, 207 and 222) that have not yet been identified either *in vitro* or *in vivo* in any species, but were clearly expressed on our microarrays, and used nCode microRNA real-time qPCR to confirm their expression in human cells. We performed a qualitative analysis of the qPCR dissociation profiles and separated PCR products by agarose electrophoresis to identify visible bands generated during the qPCR reactions. Additionally, 2 known miRNAs, miR-10a and miR-302a, were used as controls for up- and down-regulation, respectively. The PCR primers and annealing temperatures used are provided in Table 2. The differential expression of the control miRNAs obtained using the nCode real-time qPCR method agreed with both the microarray and TaqMan real-time qPCR results (Fig. 9). Furthermore, we were able to amplify 2 of the predicted miRNAs, i.e., pred-MIR191 and pred-MIR222, using primers 2 and 3, respectively, at an annealing temperature of 60°C (Table 2). The PCR amplified bands, observed for both pred-MIR191 and pred-MIR222, were cut from the gel and the sequences were then validated by cloning and sequencing of the amplicons. The mature sequences of these miRNAs were determined to be: pred-MIR191; 5′-AGCAGGTGCGGGGCGGCG-3′ and pred-MIR222; 5′-CAGTGCAAGTGTAGATGCCGA-3′. The predicted miRNAs, pred-MIR191 and pred-MIR222, were submitted to miRBase (http://www.mirbase.org/) and have been assigned the names hsa-miR-3665 and hsa-miR-3666, respectively.

**Table 2:** PCR confirmation of predicted miRNAs.

Gene	Primer	Primer Sequence 5′-3′	Anneal temp (°C)
10a	1	TACCCTGTAGATCCGAATTTGTG	57
10a	1	TACCCTGTAGATCCGAATTTGTG	58.5
302a	1	TAAGTGCTTCCATGTTTTGGTGA	57
302a	1	TAAGTGCTTCCATGTTTTGGTGA	60
pred-MIR112	1	GGACACAGTGGCACGGGGTGGC	57
pred-MIR112	2	ACACAGTGGCACGGGGTGGCAA	57
pred-MIR112	3	ACACAGTGGCACGGGGTGGC	57
pred-MIR145	1	TTTAAATGGGCCACCCTGTAAA	57
pred-MIR145	1	TTTAAATGGGCCACCCTGTAAA	58.5
pred-MIR145	1	TTTAAATGGGCCACCCTGTAAA	60
pred-MIR189	1	TGCTGGTGAGGGAGCCCTGCTG	57
pred-MIR191	1	CAGCAGCAGGTGCGGGGCGGCG	60
pred-MIR191	2	AGCAGGTGCGGGGCGGCGAAA	57
**pred-MIR191**	**2**	**AGCAGGTGCGGGGCGGCGAAA**	**60**
pred-MIR191	3	AGCAGCAGGTGCGGGGCGGCGA	60
pred-MIR207	1	ACTTTGTGCTGGTGCCGGGGAA	57
pred-MIR222	1	CAGTGCAAGTGTAGATGCCGAC	57
pred-MIR222	1	CAGTGCAAGTGTAGATGCCGAC	58.5
pred-MIR222	1	CAGTGCAAGTGTAGATGCCGAC	60
pred-MIR222	2	CAGTGCAAGTGTAGATGCCGA	57
pred-MIR222	2	CAGTGCAAGTGTAGATGCCGA	60
pred-MIR222	3	CAGTGCAAGTGTAGATGCCGAAA	57
**pred-MIR222**	**3**	**CAGTGCAAGTGTAGATGCCGAAA**	**60**
pred-MIR222	4	GTGCAAGTGTAGATGCCGACAA	57
pred-MIR222	4	GTGCAAGTGTAGATGCCGACAA	60
SLAIN1	Forward	TTCACCTCAAGCCCAAACTC	60
	Reverse	TGACTATTTCGCACGGTGAC	
FOXP2	Forward	ATTGGATGACCGAAGCACTG	60
	Reverse	GTGCAAGTGGGTCATCATTG	

Note: Primers and conditions used for sequencing of the predicted miRNAs are bolded.

Both pred-MIR191 and pred-MIR222 are associated with host genes suggesting that, potentially, their expression could be co-regulated. Thus, pred-MIR191 lies 219bp upstream of and antisense to *SLAIN1* (SLAIN motif family, member 1), a gene strongly expressed in human embryonic stem cells and down-regulated during differentiation, but expressed in the brain [45]. Similarly, pred-MIR222 is in an intron of *FOXP2* (forkhead box P2), a transcription factor expressed during embryogenesis and thought to be involved in development of motor-related circuits in mammalian brain [46]. We compared the expression of these genes at day 0 and day 8 of the RA-treatment. The results showed that both pred-MIR191 and its host gene *SLAIN1* were up-regulated after 8 days RA (Fig. 9; NT2-8D/NT2-undiff: pred-MIR191 - 2.3-fold, p0.04 and *SLAIN1*- 5.9-fold, p0.1). We also confirmed the co-expression of *FOXP2* and pred-MIR222 at day 0 and day 8 of RA (Fig. 9; NT2-8D/NT2-undiff: *FOXP2* - 2.1-fold, p0.02 and pred-MIR222 - 1.4-fold, p0.1).

## Discussion

We performed a comprehensive analysis of miRNA expression patterns during RA-induced differentiation of human NT2 cells. This well characterized cell system is a valuable *in vitro* model of neurogenesis, in which these “surrogate” stem cells undergo, in response to RA treatment, expansion of precursor pool, neural fate commitment and terminal differentiation to neurons and astroglia [32], [33], [47], [48]. Using this model we were able to identify distinct subsets of miRNAs associated with different developmental stages suggesting their role in molecular pathways critical for neurodevelopment.

### Validity of experimental model

A number of observations arising from our data would appear to support suitability of our experimental approach and the choice of NT2 cell system to study the function of miRNAs in neurogenesis. Firstly, greater than 90% of microarray expression data was validated by qPCR, indicating that the methodology used is not subject to a notable degree of false positive/negative signals. Furthermore, a number of the miRNAs we identified as induced by RA are consistent with those RA-induced miRNAs previously reported by Sempere et al. [26] (Fig. S1A). Moreover, the undifferentiated NT2 cells expressed members of ES cell specific polycistronic cluster (miR-367, miR-302a, b, c and d), consistent with their stem cell properties [29], [30], which, subsequently, were down-regulated as the RA time-course progressed and the cells acquired the neural precursor features [32], [34]. Notably, the expression of this cluster is regulated by the transcription factor *POU5F1*, which is down-regulated upon addition of RA to NT2 cells [34], [49], [50]. Finally, and most importantly, there was a very good agreement regarding miRNA data sets generated using the NT2-derived cells and human primary neurons and astrocytes, confirming that NT2 cells represent an accessible and reproducible model of human neural development.

A number of miRNAs expressed on our microarrays have been characterized in the literature and linked to the process of neurogenesis (Fig. S2) [51]. One example is miR-124, which once released from repression by *REST*, is particularly important in determining neural cell fate via regulation of Sox9 [12], [16], and which we also found to be neuronal in both NT2 and primary human neurons. Another is miR-23, which, in agreement with the report of Smirnova et al. [11], was preferentially expressed in astrocytes. Similarly, the let-7 miRNAs, reported to be prominently up-regulated in neurogenesis by Krichevsky et al. and Smirnova et al. [10], [11], showed clear neuronal expression in both NT2-N and primary human neurons. In our experiments the expression of at least two let-7 members (let-7b and let-7f) increased during the 28 day time-course. We also detected expression of a number of miRNAs associated with neurological disorders, such as miR-29 and miR-107 associated with Alzheimer's disease [20], [21].

Taken together, the changes in miRNA expression profiles observed during RA-induced differentiation of NT2 cells could be considered to reflect their involvement in different stages of human neurodevelopment. Therefore, NT2 cells represent a convenient experimental system amenable to future mechanistic studies required to elucidate specific functions of miRNAs in neurogenesis.

### Implications for neurodevelopment

A preliminary analysis of the microarray data revealed an increase in the number of miRNAs expressed as the RA time-course progressed (Fig. 2), in agreement with the progressive, non-restrictive model of miRNA expression in which there is a trend to increased miRNA expression signature with developmental state [52]. We have identified two subsets of miRNAs with potential functional significance for the process of neurogenesis. These were 16 miRNAs that were up-regulated early in the RA time-course and which, in all cases but one, peaked in expression between day 6 and 14 of RA treatment (Fig. 5) and 18 miRNAs, which were induced between 8 and 12 days of RA (Fig. 8). We proposed that these subsets were of particular biological significance, as these would act early in the differentiation process and might play a role in cell-fate determination and maintenance of differentiated state as they were expressed prior to, and during the cell cycle exit of neural precursors, respectively [32].

In the first 8 days of the NT2-RA time-course, *REST* is down-regulated, allowing the expression of a number of neuronal genes and committing cells to a neural phenotype [34], [53]. Although miR-9 has been shown to down-regulate *REST* via MREs in the *REST* 3′-UTR it is also repressed by *REST* [16], [19]. Since, in our NT2-RA time-course, the decrease in *REST* expression preceded the increase in expression of miR-9 we investigated whether other miRNAs, which increased in the first 8 days, may target *REST* (Fig. 5). We identified a number of miRNAs with conserved MREs (Fig. 5). Perhaps the most interesting was a highly conserved MRE in the *REST* 3′-UTR for miR-219-5p, the most strongly up-regulated of all miRNAs during the first 8 days of RA treatment. This miRNA is expressed early in development, and its dysregulation leads to embryonic defects in zebrafish [54]. Furthermore, using miRanda [40], we identified an MRE for dre-miR-219 in the zebrafish genomic sequence (danRer5), approximately 3 kbp downstream of the *REST* coding region (i.e. within the putative zebrafish *REST* 3′-UTR). Taken together this suggests an important role for this miRNA in development that may involve the regulation of *REST*. We observed a large increase in the number of miRNAs expressed at 12 days RA and many of these miRNAs were significantly up-regulated between 8 and 12 days including the *REST* regulated miRNAs miR-9 and miR-124, which play important roles in neurogenesis [9], [10], [14], [16] and miR-21, which has been proposed as a suppressor of pluripotency in embryonic stem cells [41] (Fig. 8). Using a combination of data from a genome-wide ChIP-Seq analysis for *REST* in human K562 cells and predicted sites from the human RE-1db (http://www.bioinformatics.leeds.ac.uk/RE1db_mkII/) [42], [55], [56] we identified a number of other candidate *REST* regulated miRNAs (Fig. 8 and Table 2). With the exception of miR-301, all of these putative *REST* target miRNAs were found to be expressed in both NT2-N and PHN, and many of them were differentially expressed between neurons and astrocytes suggesting that they may be regulators of neuronal development and function (Fig. 8).

Previously our group has characterized gene expression patterns during the RA induced neurogenesis of NT2 cells and identified sets of genes considered significant for cell cycle regulation and differentiation [34]. Here we observed that many of these differentiation related genes were predicted targets for a number of the miRNAs up-regulated during this time frame based on a comparison of our miRNA profiles with the Microcosm Targets database (http://www.ebi.ac.uk/enright-srv/microcosm/htdocs/targets/v5/). Whilst these target sites are predicted and currently remain un-tested, the comparisons between the mRNA and miRNA expression data sets may provide a fast screening method for selection of candidate miRNA/target pairs for further direct functional analysis. For example, a number of HOX genes that are down-regulated during neurogenesis contain predicted binding sites for miRNAs expressed after 12 days of RA treatment. Interestingly, the interaction between the neuronal miR-10a and *HOXA1* gene, has already been experimentally verified [57]. Cell cycle exit is also characterized by a decrease in the expression of cyclins [34]. Therefore, miRNAs expressed after 12 days RA, particularly those expressed in post-mitotic neurons, such as miR-10a, 10b, 20a, 181a and let-7b, which are predicted to target cyclin genes, would be excellent candidates for further functional analyses. One of these miRNAs, let-7b, has recently been shown to target cyclin genes D1, D3, and A1, and cyclin-dependent kinase *CDK4*, *in-vitro* [58].

The comparisons of expression profiles between human primary and NT2-derived cells revealed miRNAs that might play a role in the development and maintenance of differentiated neurons and astrocytes. Thus, miRNAs expressed in primary human astrocytes showed different expression profiles to miRNAs expressed in primary human neurons and during the differentiation and maturation of NT2 cells. Notably, far fewer miRNAs were expressed in astrocytic than in neuronal cells suggesting that a higher complexity of gene expression regulation exists in post-mitotic neurons than in proliferation competent astrocytes. This is consistent with previous reports suggesting that larger numbers of miRNAs are expressed in differentiated and higher specialized cells such as neurons [51], [52]. The miRNAs found to be expressed in both astrocytes and neurons are perhaps involved in maintenance of the terminally differentiated state in both cell types. For example, miR-24, which we found up-regulated early during the differentiation process and subsequently expressed in both astrocytes and neurons, is known to target dihydrofolate reductase (*DHFR*) [59], a gene critical for DNA synthesis and replication; suggesting that miR-24 may play a role in cellular differentiation via targeting of *DHFR* to inhibit DNA replication. This, again, is consistent with the reported down-regulation of *DHFR* in terminally differentiated neurons [60].

### Validation of predicted miRNAs

One impediment to successful amplification of the predicted miRNAs is that the exact position and length of the mature product within the pre-miRNA is not known, and cannot be accurately predicted using currently available tools. The predicted mature miRNA sequences identified by Xie et al. [35] were determined computationally and all of their mature miRNAs were reported as 22 nt sequences. A number of the pre-miRNA and mature miRNA sequences appearing in miRBase differ from those predicted by Xie et al. [35] and in fact the mature sequence of many other miRBase entries have been modified and updated as new data is provided. Another impediment is that successful amplification with the nCode system sometimes requires modifications to the miRNA specific forward primer to improve amplification, including removing bases to lower Tm and/or adding 3′-adenines to improve specificity. It was for this reason that we also used amplicon sequencing, and from this combination of data we derived the mature sequences. The amplicon sequences of both predicted miRNAs differed from those reported by Xie et al. [35]. Pred-MIR191 was determined to be 18 nt, omitting 4 nt at the 5′ end and pred-MIR222 was 21 nt, missing 1 nt at the 3′ end. In addition we also tested the expression of the predicted miRNAs, pred-MIR191 and pred-MIR222, and the associated host genes, *SLAIN1* and *FOXP2*, and demonstrated that they followed the same kinetics. This, in turn, raised an interesting possibility that the region between *SLAIN1* and pred-MIR191, which contains both a putative promoter and a CpG island, may be responsible for co-regulation of these genes through a bi-directional promoter mechanism [61]. Furthermore the presence of a predicted binding site for pred-MIR191 in the 3′-UTR of *SLAIN1* also raises the possibility that this miRNA can regulate the translation of its host gene, a mechanism that has been proposed for miR-186 in dog [62] and for miR-763 in human, mouse and rat [63].

### Conclusions

We present what is currently the most comprehensive analysis of miRNA expression using a reproducible experimental model of human neural differentiation, upon which the further mechanistic studies required to establish the roles of miRNAs during neural development can be performed. This knowledge, in combination with identification of specific miRNA targets, will ultimately reveal the metabolic pathways that they control and bring about better understanding of molecular mechanisms controlling the process of neurogenesis.

## Materials and Methods

### Cell Culture

#### Time-course of retinoic acid treatment

Human embryonal teratocarcinoma NTera2/D1 (NT2) cells (Stratagene, La Jolla, CA) were cultured in high glucose Dulbecco's modified Eagle's medium (HG/DMEM, Invitrogen, Burlington, Ontario, Canada) supplemented with 10% fetal bovine serum (FBS, Wisent, Saint-Jean, Quebec, Canada) and differentiated with all-trans retinoic acid (RA, Sigma, Oakville, Ontario, Canada) as previously described [33]. Briefly, cells were seeded at a density of 2×106 cells per T-75 flask and treated with 10µM RA for 28 days added with fresh medium every two days. Cells were harvested for RNA extraction at 0, 2, 4, 6, 8, 12, 14, 21, and 28 days of the RA treatment. The time-course experiments were repeated several times to provide multiple biological replicates for microarray analysis.

#### Generation of NT2-N neurons and NT2-A astrocytes

Pure NT2-N neurons were isolated from 28-day RA-treated cultures as previously described [44]. Briefly, the RA-treated cells were trypsinized, re-plated at a 1∶6 dilution in new T-75 flasks and cultured for 9 days in RA-free HG/DMEM supplemented with 5% FBS and mitotic inhibitors (1µM cytosine β-D-arabinofuranoside, 10µM uridine, and 10µM 5-Fluoro-2′deoxyuridine). The NT2-N neurons, growing on the top of underlying astrocytes (NT2-A), were separated by gentle trypsinization (0.015% v/v) and mechanical shake-off. The neurons were re-plated on poly-D-lysine and Matrigel-coated T-25 flasks (2×10^6^ cells/flask) and maintained for an additional 7 days in a 50∶50 mix of NT2-A -conditioned medium and fresh DMEM with 10% FBS before being harvested for RNA extraction.

Pure NT2-A astrocytes were also generated from 28-day RA-treated cultures after the removal of neurons. Briefly, the RA-treated cells were grown overnight in RA-free HG/DMEM supplemented with 5% FBS and weakly attached neurons were removed by gentle trypsinization (0.015% v/v) and mechanical shake-off leaving the astrocytic cells in the flask. This step was repeated and the remaining NT2-A cells were maintained in HG/DMEM and 5% FBS with medium changed twice a week. Cells were subcultured when confluent. Total RNA was extracted from 2 to 8 weeks old NT2-A cultures.

*Primary human neurons and astrocytes*. Fresh primary human neurons (PHN) from 21 week fetuses were obtained from ScienCell Research Laboratories (Carlsbad, CA) and were directly pelleted for RNA extraction.

Frozen primary human fetal astrocytes (PHAf) from 20–21 week fetuses were received from ALLCELLS (Emeryville, CA) and were cultured in Astrocyte Medium provided by the company.

Frozen primary human embryonal astrocytes (PHAe) from 9–10 week embryos were received from StemCell Technologies (Vancouver, BC, Canada) and were cultured as recommended by the supplier and harvested for RNA extraction. Cell populations were expanded and then harvested for RNA extraction.

All of the human cells used in this study were obtained in accordance with principles embodied in the Declaration of Helsinki (Code of Ethics of the World Medical Association).

### RNA Extraction

Total RNA was isolated from the cells using TRI-Reagent (Molecular Research Center, Cincinnati, OH) according to the manufacturer's instructions. High molecular-weight RNAs were removed by precipitation with 12.5% PEG-8000 and 1.25 M NaCl using the method of Thompson et al. [64].

### Microarray Platform

Oligonucleotide probes for 405 miRNAs, synthesized by MWG-Biotech (High Point, NC) and Sigma-Genosys (Saint Louis, MO), were resuspended in Nexterion Spot II buffer (Schott Nexterion, Louisville, KY) at a concentration of 40µM and printed in triplicate onto Nexterion Slide E epoxy-coated glass slides with an Affymetrix 417 Arrayer (Affymetrix, Santa Clara, CA) in a pattern of four 20×18 sub-arrays with 350µm spacing between spots. The spotted “trimeric concatemer” probes were complementary to the mature miRNA sequences concatenated three times, such that a 22 nt miRNA was represented on the array by a 66 nt probe. Sequences for 210 human and 49 mouse miRNAs were obtained from miRBase version 5.1. All miRNA names and accession numbers were updated to miRBase 14.0 (Fig. S1A). Sequences for an additional 146 computationally-predicted miRNAs were obtained from Xie et al. [35]. Of these, 36 were subsequently identified as human miRNAs in miRBase version 14.0 (Fig S1A). Probes for U6 RNA and five human and two mouse tRNAs were spotted as positive controls. Negative controls were three sequences with no sequence similarity to any known mammalian miRNAs [65]. The M13-reverse universal sequencing primer was used as a control for specificity of hybridization. For intra-assay validation, the standard deviation calculated for miRNA triplicates was <10% for the vast majority (>99%) of oligos. Furthermore, we collected NT2 cells from several independent experiments such that most time-points in the neural differentiation time-course represented at least three biological replicates which were averaged to arrive at the final expression levels reported. 25 µg of RNA was utilized for the time-course experiments, and 10 µg for the neuron and astrocyte experiments.

### RNA Labeling and Microarray Hybridization

RNA labeling and hybridization to the microarrays was carried out using a method adapted from Thompson et al. [64]. Briefly, 10 or 25 µg total RNA was PEG-precipitated (as above), resuspended in 6 µl water, and mixed into a labeling reaction (final volume 10 µl) consisting of 500 ng 5′-phophate-cytidyl-uridyl-Cy3-3′ (Dharmacon, Chicago, IL), 10% DMSO, 0.1 mM ATP, 50 mM HEPES (pH 7.8), 3.5 mM DTT, 20 mM MgCl_2_, 0.1 µg BSA, and 20 U T4 RNA ligase (NEB, Ipswitch, MA). Reactions were incubated on ice in the dark for 2 hours before precipitation with 0.27 M sodium acetate, 20 µg glycogen, and 2.7 volumes ethanol in a 200 µl final volume. Following 10 min incubation on ice and 10 min centrifugation, the pellet was washed in 70% ethanol, centrifuged 5 min, briefly dried, and resuspended in 6 µl water. Labeled RNA samples were hybridized to microarrays in a 35 µl final volume composed of 400 mM Na_2_HPO_4_ (pH 7.0), 5% SDS, 0.8% BSA, and 12% formamide. Hybridization mixtures were denatured at 95°C for 4 minutes and immediately pipetted onto glass slides covered with 22×25 mm LifterSlips (Erie Scientific Company, Portsmouth, NH). Hybridizations were performed in custom-made sealed chambers submerged in a 37°C water bath overnight (18 hours). Slide washes were 3 min long: once in 2× SSC/0.025% SDS, three times in 0.8× SSC, and twice in cold 0.4× SSC. Immediately following the last wash, slides were dried by centrifugation and scanned with a ScanArray 5000XL (Packard BioScience, Meriden, CT) confocal scanner at 10 µm resolution.

### Microarray Data Analysis

Microarray scans were analyzed with QuantArray software version 3.0 (Packard BioScience, Meriden, CT) using an adaptive quantitation method. Spots of poor quality on the arrays were flagged for removal from the data processing. Cy3 mean pixel intensities were background subtracted, log transformed, and replicate probes on the arrays were averaged. The intensities of each unique probe, including positive and negative controls, were normalized by subtracting the median log2 intensity of all unique probes on the array, thus median centering all of the arrays to zero. miRNAs were considered expressed if their normalized log2 intensity was ≥2. Differentially-expressed miRNAs were identified using a SAM-FDR (Significance Analysis of Microarrays-False Discovery Rate [66] threshold of 5% and a fold-change of 2 in either direction. The microarray data are MIAME compliant and have been deposited in the Gene Expression Omnibus (GEO; http://www.ncbi.nlm.nih.gov/geo) under accession GSE15888.

### Real-time qPCR of known miRNAs

Real-time qPCR assays for known miRNAs were carried out using the TaqMan® Human MicroRNA Assay (Applied Biosystems, Foster City, CA). Briefly, 10 ng total RNA was reverse transcribed in 15 µl reactions containing 3.33 U MultiScribe reverse transcriptase, 0.25 U RNase inhibitor, 1 mM dNTPs, and 3 µl of the provided miRNA-specific looped RT primer. Reactions were incubated at 16°C for 30 min, 42°C for 30 min, and 85°C for 5 min. PCR reactions of 20 µl were prepared with 1.4 µl of the RT reaction product, TaqMan 2× Universal PCR Master Mix, and the provided TaqMan MicroRNA Assay mix containing pre-formulated forward and reverse primers and the FAM-linked TaqMan MGB probe. Assays were run in triplicate on an ABI Prism 7000 SDS thermocycler as follows: 95°C for 10 min, and 40 cycles of 95°C for 15 s and 60°C for 1 min. All assays were repeated at least twice. miRNA expression changes were considered confirmed if the qPCR showed fold changes greater than 2 in the correct direction and weak if the qPCR fold change was less than 2, but in the correct direction.

### Real-time qPCR, cloning and sequencing of predicted miRNAs

Real-time qPCR assays for predicted miRNAs and positive controls was performed using the NCode™ GreenER™ miRNA First-Strand cDNA Synthesis and qRT-PCR Kit (Invitrogen, Burlington, Ontario, Canada) as described by the manufacturer, using 1µg of total RNA. Assays were run on an ABI Prism 7000 SDS thermocycler as follows: 50°C for 2 min, 95°C for 10 min, and 40 cycles of 95°C for 15 s and 57 or 60°C for 1 min. Primers and annealing temperatures were as shown in Table 2. Ncode PCR reactions were resolved by 4% agarose gel electrophoresis and amplified products purified using the Geneclean Spin Kit (MP Biomedicals, Solon, OH). The purified PCR amplicons were then ligated into TOPO TA vectors (Invitrogen, Burlington, Ontario, Canada) and subjected to DNA sequencing using the M13 reverse primer at our in house facility.

### Real-time qPCR of predicted miRNA host genes

Real-time qPCR assays for miRNA host gene mRNA was carried out using the SuperScript™III Platinum® SYBR® Green One-Step qRT-PCR Kit (Invitrogen, Burlington, Ontario, Canada) as described by the manufacturer, using 1µg of total RNA. Assays were run on an ABI Prism 7000 SDS thermocycler as follows: 50°C for 3 min, 95°C for 5 min, and 40 cycles of 95°C for 15 s and 60°C for 30 s. Primers were as shown in Table 2.

### Target Prediction of predicted miRNAs

3′-UTR regions of genes from the human genome (hg17) were obtained using the UCSC genome browser (http://genome.ucsc.edu/) [67]. The human *REST* 3′-UTR sequence was obtained from Packer et al., 2008 [19] and the mouse and dog sequences were derived from the human sequence and obtained from the UCSC genome browser using the Vertebrate Multiz Alignment & Conservation track [67]. The miRNA binding site prediction program miRanda [40] was installed locally and used to search for miRNA target sites with the same parameters as used for miRNA target prediction at microRNA.org (Score cutoff S> = 140, Energy cutoff E< = −7.0, Gap opening: −9.0, Gap extension −4.0, 5′ scaling: 4).

## Supporting Information

Figure S1 Expression profiles of miRNAs. A. Summary table of miRNAs expression and analysis. Complete expression profiles for all miRNAs are presented in heatmap format. In addition, specific comparisons were also performed. The figure indicates: (i) miRNAs up- or down-regulated during the time course of RA treatment based on the comparative analysis of NT2-undiff vs. NT2-8D, NT2-8D vs. NT2-12D and NT2-undiff vs. NT2-28D (labeled as Up and Down with respect to the earlier time-point); (ii) miRNAs differentially expressed during terminal differentiation of NT2 cells to neurons and astrocytes as reflected by the comparisons of NT2-N vs. NT2-28D and NT2-A vs. NT2-28D (labeled as Higher or Lower with respect to NT2-28D); (iii) neuronal and astrocytic miRNAs (labeled as Neuronal or Astrocytic) were designated based on their expression levels in NT2-A vs. NT2-N and in primary human neurons vs. primary astrocytes (PHN vs. PHAf and PHN vs. PHAe ). In all figures, the intensity of the yellow scale in the heat map corresponds to the mean log2 expression of miRNAs on the microarrays, from zero and lower (black) to eight and higher (bright yellow). B. Expression profiles of miRNAs differentially expressed in NT2 cells during the RA time course. The figure shows miRNAs up- or down-regulated during the RA time course based on the comparisons of NT2-undiff vs. NT2-8D, NT2-8D vs. NT2-12D, and NT2-undiff vs. NT2-28D (labeled as Up and Down with respect to the earlier time-point). C. Expression profiles of miRNAs in terminally differentiated NT2 cells. miRNAs differentially expressed during the terminal differentiation of NT2 cells to neurons and astrocytes as reflected by the comparisons of NT2N vs. NT2-28D and NT2A vs. NT2-28D (labeled as Higher or Lower with respect to NT2-28D). miRNAs were classified as neuronal or astrocytic based on their expression levels in NT2-A vs. NT2-N. D. Expression profiles of astrocytic and neuronal miRNAs. miRNAs classified as neuronal or astrocytic based on their expression levels in NT2-A vs. NT2-N as well as in primary human neurons and fetal or embryonic astrocytes (PHN vs. PHAf and PHN vs. PHAe).
(0.12 MB XLS)

Figure S2 Expression patterns of miRNAs associated with the central nervous system. The list of miRNAs and the information regarding their reported functions in the CNS were taken from a recent review of Zeng [51]. The figure shows their expression patterns during RA-induced differentiation of NT2 cells (up- or down- regulated in NT2-undiff vs. NT2-8D, NT2-undiff vs. NT2-28D and NT2-8D vs. NT2-28D) and in NT2-N neurons and NT2-A astrocytes (higher or lower in NT2N vs. NT2-28D and NT2A vs. NT2-28D). miRNAs were classified as neuronal or astrocytic based on their expression levels in terminally differentiated NT2 cells (NT2-A vs. NT2-N) as well as in primary human neurons and fetal astrocytes (PHN vs. PHAf ) or embryonic astrocytes (PHN vs. PHAe).
(0.03 MB XLS)

Table S1 Real-time qPCR validation of microarray results.
(0.03 MB XLS)
